# Beyond the Chicken: Alternative Avian Models for Developmental Physiological Research

**DOI:** 10.3389/fphys.2021.712633

**Published:** 2021-10-21

**Authors:** Josele Flores-Santin, Warren W. Burggren

**Affiliations:** ^1^Facultad de Ciencias, Biologia, Universidad Autónoma del Estado de Mexico, Toluca, Mexico; ^2^Developmental Integrative Biology Research Group, Department of Biological Sciences, University of North Texas Denton, Denton, TX, United States

**Keywords:** embryo, development, chicken, animal model, domestication, bird

## Abstract

Biomedical research focusing on physiological, morphological, behavioral, and other aspects of development has long depended upon the chicken (*Gallus gallus domesticus*) as a key animal model that is presumed to be typical of birds and generally applicable to mammals. Yet, the modern chicken in its many forms is the result of artificial selection more intense than almost any other domesticated animal. A consequence of great variation in genotype and phenotype is that some breeds have inherent aberrant physiological and morphological traits that may show up relatively early in development (e.g., hypertension, hyperglycemia, and limb defects in the broiler chickens). While such traits can be useful as models of specific diseases, this high degree of specialization can color general experimental results and affect their translational value. Against this background, in this review we first consider the characteristics that make an animal model attractive for developmental research (e.g., accessibility, ease of rearing, size, fecundity, development rates, genetic variation, etc.). We then explore opportunities presented by the embryo to adult continuum of alternative bird models, including quail, ratites, songbirds, birds of prey, and corvids. We conclude by indicating that expanding developmental studies beyond the chicken model to include additional avian groups will both validate the chicken model as well as potentially identify even more suitable avian models for answering questions applicable to both basic biology and the human condition.

## Introduction

Animal models have long been extensively employed in biomedical research—for an entry into the substantial literature, see Bähr and Wolf (2012), Andersson (2016), Bolker (2017), Andersen and Winter (2019), and Robinson et al. (2019). Birds of all developmental stages play an important role in biomedical research and have provided major insights into processes in development (Bolin and Burggren, 2013; Nowak-Sliwinska et al., 2014; Burggren et al., 2016; Towers, 2018; Burggren and Rojas Antich, 2021), aging (Holmes, 2004; Swanberg et al., 2010; Austad, 2011), physiology (Vilches-Moure, 2019; Williams et al., 2020), immunology (Davison, 2003; Kohonen et al., 2007), infectious and other diseases (Hawkridge, 2014; Wang and Wang, 2016), and pharmaceutical testing (Datar and Bhonde, 2011; Bjornstad et al., 2015; Wu et al., 2018), to name just a few studies in just a few of the many disciplines that have exploited and benefitted from avian models.

**Figure 1:**
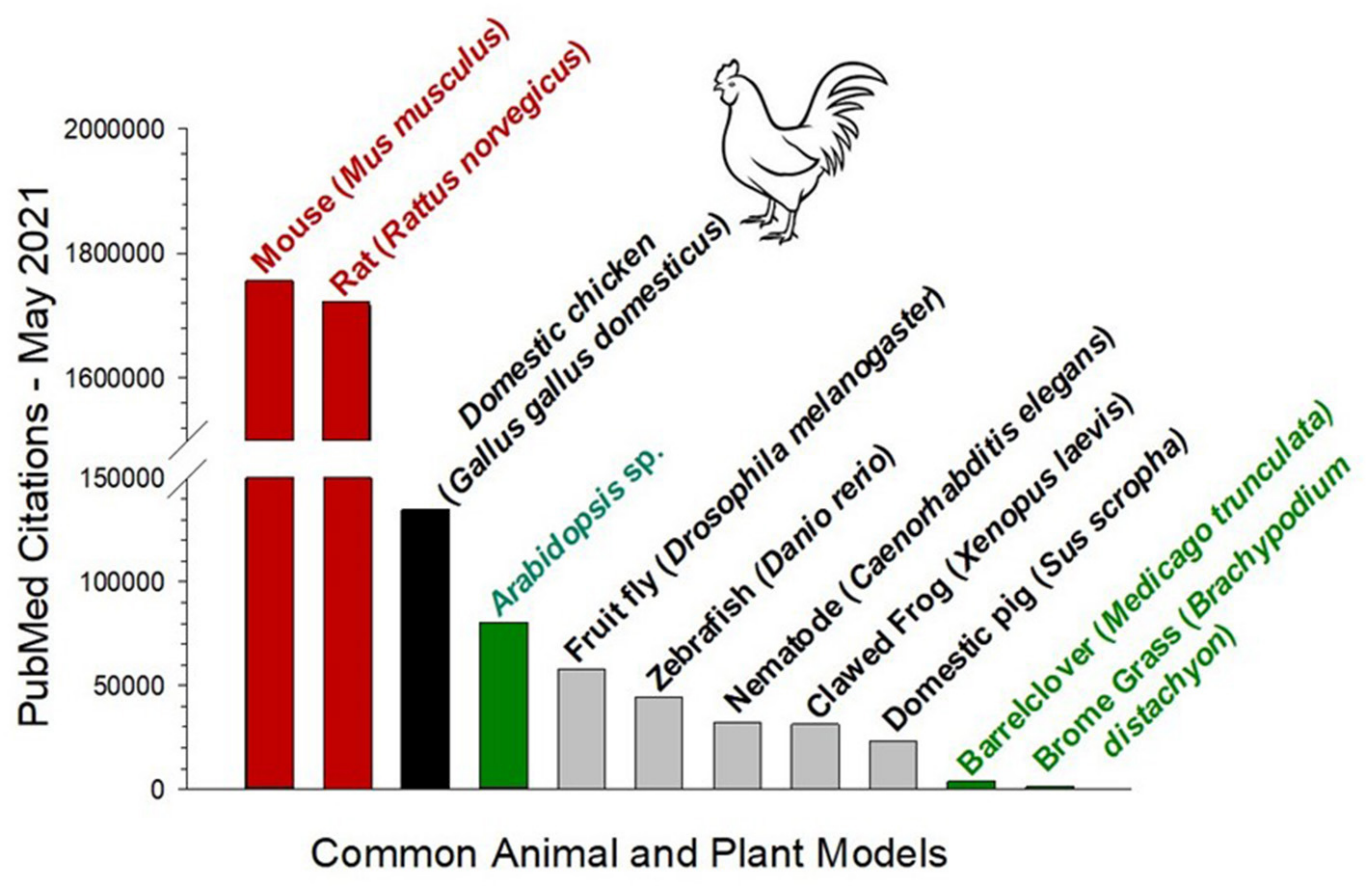
Total number of PubMed citations of common animal and plant models derived from the Latin genus and species. Data acquired May, 2021. The two common rodent models (red) are highly dominant in biomedical research. However, while the zebrafish *D. rerio* and the nematode *C. elegans* are receiving much attention, both have less than a third of the PubMed citations as the domestic chicken (black). Also shown for comparison are three commonly used plant models (green).

Central as a bird model in biomedical research, especially in developmental studies, has been the domestic chicken *Gallus gallus domesticus* (Figure 1). The value of this venerable animal model cannot be overstated. Selective breeding has led to breeds with characteristics of particular interest to biomedical investigation. A clear example is the inadvertent development chicken breeds that are hypertensive and or hyperglycemic (Julian, 1998; Ji et al., 2012; Khajali and Wideman, 2016; Matos et al., 2018; Lake and Abasht, 2020). Additionally, the advent of genetic editing has produced a new wave of chicken and other models that are further accelerating their use in biomedical research (Lee et al., 2015; Davey et al., 2018; Sid and Schusser, 2018; Chojnacka-Puchta and Sawicka, 2020; Koslová et al., 2020). As a consequence, the chicken and especially the chicken embryo have had a huge influence on developmental physiology, not only for understanding basic process in physiological development, but also in the important task of modeling human disorders.

However, in this perspective we suggest that expanding studies of embryos to adults *beyond* the chicken model to include additional avian species may result in even deeper insights into both fundamental questions in basic biology and the human condition. This is not an original thought, for nearly a decade ago Bolker's article title offered the warning “*There's more to life than rats and flies”* (Bolker, 2012). To paraphrase her title, we suggest that there's more to avian research than *G. gallus domesticus*, as we explore in this perspective focusing on research in developmental biology.

Before exploring alternative avian models in developmental research, however, we first briefly consider how certain animal models have been used in developmental and other forms of research—in fact have come to dominate such research—and explore what actually comprises a useful animal model.

## Dominant Animal Models—an Potential Handicap for Biomedical Research?

The emergence of an animal model is often based on a series of favorable characteristics of that model, with convenience and convention being two of the most important. Yet, as Bolker succinctly states “*dominant models may bias research directions*” (Bolker, 2017). Indeed, the ultimate success of the model often involves aspects of a self-fulfilling prophecy. Thus, the more the model is employed in research, the greater is the body of knowledge available of the model, and so the more useful it becomes—which in turn leads to greater use of the model, more knowledge and more usefulness, etc. Once a model is established, entire communities may spring up around the model—e.g., the worm community using *Caenorhabditis elegans*, the fly community using *Drosophila melanogaster*, the zebrafish community using *Danio rerio*, etc. Ironically, because of the success of established models, potentially even more suitable, more relevant animal models may be largely overlooked or ignored because of their perceived marginal position in animal research. A classic example is the zebrafish *D. rerio* and the community that formed around it. Recognized as a genetically tractable system in the early 1980s (Nüsslein-Volhard, 2012), the zebrafish embryos, larvae, and adults are to this day advanced as a model because of its fecundity, rapid growth, transparent embryos, etc. Zebrafish were discovered as a genetic model because they happened to be available in Tübingen, Germany and were quickly recognized as potentially of great use. The rest is history, as they say, with PubMed now listing nearly 45,000 papers over the last 75 years containing the word “zebrafish”! Clearly, this is an animal model of critical importance to both basic and biomedical research because of a somewhat serendipitous discovery. Yet, considering that there are ~30,000 species of teleost fishes, it is possible—even likely—that an even more tractable, effective model than zebrafish potentially exists, though such a model is unlikely to ever gain traction in the zebrafish or any other research community. Notably, the notion of looking for the most suitable animal model to answer a basic question is neither new nor novel. In 1929 Nobel prize winner (1920) August Krogh stated “*For many problems there is an animal on which* (a physiological problem) *can be most conveniently studied”* (Krogh, 1929). Indeed, the so-called “August Krogh principle” is one of the guiding principles in animal physiology (Krebs, 1975; Burggren, 1999, 2020; Strange, 2007).

Given these general perspectives on animal models, we ask the question “*Is the very widely employed chicken model—essentially the ‘go to' model for avian-based research in developmental physiology and many other areas—the best model for every biomedical research project exploiting an avian model*”? Our answer to this question is “*No…. and yes.”* Certainly, it is not our intention to be highly critical of the chicken as an animal model for development. Essentially, every animal model has many important attributes that make it a useful model in the first place, but also possesses characteristics that potentially obfuscate the experimental results when comparting them to other animals or to humans. Rather, we are critical of biomedical research that narrowly uses only the chicken as an avian model to the exclusion of other species that could actually be more tractable in testing hypotheses and answering questions.

## Pros and Cons of the Chicken as an Avian Experimental Model

The huge number of tons of chicken meat and eggs produced globally every year (FAO, 2020) are only possible as a result of the many traits and characteristics that make the chicken one of the world's most effective production animals (FAO Production Statistics, 2021). But these traits could also make it a complicated animal model, whether as embryo of adult. The main historical reasons for the creation of chicken breeds have been meat production, egg production, game (cock fighting), and ornamental use. With around 500 different breeds distinguished by feather and/or skin color, number of toes, size, comb shape/color, feathering pattern, and place of origin, the chicken is often considered the vertebrate with the highest number of artificially selected breeds (Crossley and Altimiras, 2012; Roth and Lind, 2013; Bílková et al., 2017). More specifically, different breeds are variously highly efficient at converting feed into muscle, having resistant to cold temperatures or resistant to heat, laying eggs at high frequency, having uncommon feathering patterns, and so on. Given this diversity of chicken breeds and their specialization, it is difficult to pick a specific breed that could encompass the whole of possible biological responses of the chicken. Therefore, we posit that it is important to understand what led to modern chickens and what that means for developmental and other biomedical research, as we now explore.

### What Is the Modern Chicken—and Why Does It Matter to Developmental Research?

#### Origins of the Domestic Chicken

The chicken (*G. gallus domesticus*) has been the subject of intense selection, and indeed is one of the most highly selected of all domesticated species, perhaps only rivaled by canines (Vilà et al., 1999; Leroy, 2011). It is not our intent to review this subject in detail, as a comprehensive review on the origin, distribution, adaptation, and evolution of the domestic chicken has recently appeared (Liu et al., 2006; Rubin et al., 2010). Briefly, the domestic chicken has several physiological, morphological and behavioral differences from the ancestral red jungle fowl (*G. gallus*), the bird presumed to be the basis for the modern domestic chicken. The general consensus emerging from genomic investigations into the past evolutionary history of the modern chicken is that the domestic chicken (with all its breeds) originated in Asia (Lawal and Hanotte, 2021). Red jungle fowl range from southeast to south Asia, with populations of a range of subspecies still existing in the wild (Al-Nasser et al., 2007). There is still debate as to which country first domesticated the red jungle fowl, but the beginning of the process is agreed to be in the early 1800's. The red jungle fowl shares many traits with the modern chicken, further supporting the claim of origin of chickens in jungle fowl (Rubin et al., 2010). However, recent molecular evidence suggests that the modern chicken is most probably a hybrid occurring between red jungle fowl, green jungle fowl (*Gallus varius*), gray jungle fowl (*Gallus sonneratii*), and the Srilankan jungle fowl (*Gallus lafayetii*) (Table 1) (Liu et al., 2006; Al-Nasser et al., 2007; Eriksson et al., 2008; Rubin et al., 2010; Roth and Lind, 2013). These observations on the origin and evolutionary history are aided by extensive genomic analysis of the chicken, in which more than 14,000 quantitative trait loci (QTL) representing ~450 different traits garnered from ~350 publication have been identified (https://www.animalgenome.org/cgi-bin/QTLdb/GG/index, accessed July 23, 2021).

**Table 1:** Taxonomy of the chicken.

**Class**	**Aves**
Order	Galliformes
Family	Phasianidae
Subfamily	Phasianidae
Genus	*Gallus* (Brisson, 1760)
Species	*Gallus gallus* (Linnaeus, 1758)
Subspecies	*Gallus gallus gallus* (Linnaeus, 1758)
Subspecies	*Gallus gallus spadiceus* (Bonnaterre, 1792)
Subspecies	*Gallus gallus bankiva* (Temminck, 1813)
Subspecies	*Gallus gallus marghi* (Robinson and Kloss, 1920)
Subspecies	*Gallus gallus jabouillei* (Delacour and Kinnear, 1928)
Subspecies	*Gallus gallus domesticus*
Subspecies	*Gallus gallus gallina*
Subspecies	*Gallus gallus micronesiae*
Subspecies	*Gallus gallus philippenisis*
Species	*Gallus varius* (Shaw, 1798)
Species	*Gallus sonneratii* (Temminck, 1813)
Species	*Gallus lafayetii* (Lesson, 1831)

*Modern chicken (Gallus gallus domesticus) is shown along with the possible genetic donors and the wild sub species of the red jungle fowl (Al-Nasser et al., 2007)*.

Interestingly, recreational activities (e.g., cock fighting) is one of the proposed first uses for chickens that might have led to their domestication, with meat and egg production subsequent to their breeding for entertainment (Liu et al., 2006; Lawal and Hanotte, 2021). The development of modern breeds as food sources was then carried on with the existing genetic pool of the chicken. This selection of traits generated three groups of the domestic chicken: indigenous village chicken, the fancy chicken breeds and, in a highly dominant position, the commercial lines, with highly divergent characteristics—consider bantams and broilers, for example.

#### Concealed “Aberrant” Physiology?

**Figure 2:**
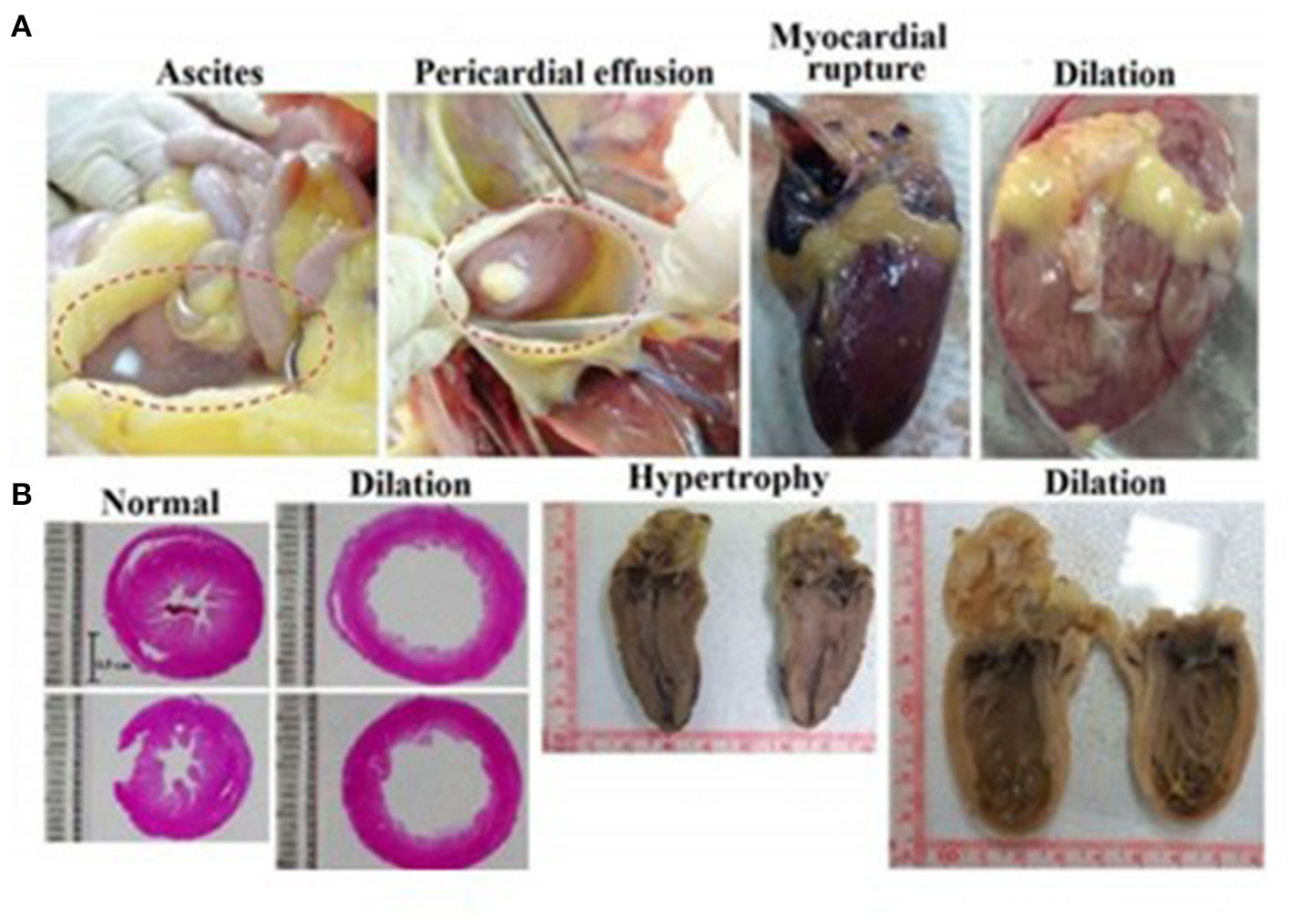
Obesity-associated cardiac disorders in adult broiler chickens. Broiler chickens 49 weeks or older fed *ad libitum* for 70 days developed significantly more cardiac lesions than calorie restricted birds. **(A)** Diet-induced alterations in cardiac gross morphology in the form of ascites (fluid accumulation), pericardial effusion, myocardial rupture, and cardiac dilation **(B)** Internal compensatory responses lead to myocardial dilation or hypertrophy as evident in hens experiencing sudden death. Not shown is the many-fold increase in collagen content in the cardiac tissue in the *ad libitum* vs. restricted diet population evident in both surviving birds and those experiencing sudden death. *Ad libitum* birds additionally showed a significantly higher incidence of arrhythmia as well as chronic elevation of systolic blood pressure (from Chen et al., 2017).

Intensive selective breeding has obviously been carried out for favorable traits such as meat and egg production. Yet, such intensive selection has led to loss of genetic diversity and has led to aberrant physiological responses not readily apparent from casual observation (Lawal and Hanotte, 2021). Such traits, which could begin with embryos and extend to adults, might not be broadly representative of chickens or even birds, generally. The broiler chicken is one of the most extreme examples of trait selection, with selective breeding yielding an increase in body mass of ~300% in the last 60 years (Cueva et al., 1974; Kamran et al., 2008; Anjola, 2016). The broiler not only has large body mass, but also presents rapid growth, making it an excellent producer and also potentially of interest to developmental biologists. However, the breeding programs that led to the modern broiler also created disadvantageous traits. For example, adult broilers are typified by cardiac disorders that are both morphological (ascites, myocardial rupture, cardiac dilation) and physiological (arrythmias, hypertension) (Figure 2), the forerunners of which may exist back to the embryos. Additionally, limb deformities and lameness are prevalent among these birds. Some researchers attribute this problem in broilers to industry standards in breeding while also acknowledging a genetic factor for leg weakness (Zubair and Leeson, 1996; Knowles et al., 2008). Moreover, while broiler chickens are most often forwarded as the example of pathophysiological states that can emerge from intensive breeding, layer chickens as well are prone to pathophysiologies such as avian osteoporosis associated with the intense burden of providing large amounts of calcium associated with egg shell formation (Webster, 2004; Whitehead, 2004). Such pathophysiologies emerge as fractures of the keel and leg bones (Webster, 2004; Toscano et al., 2020; Wei et al., 2020).

#### Is There a “Wild Type” Domestic Chicken as a Basis for Comparison?

The comparison of the chicken with other animal models generates an interesting observation—there is no longer a wild type breed in chickens. The closest to a wild type could be the red jungle fowl, which is considered only one of the parental species to the hybrid modern chicken. Other animal models such as mice, rats, the nematode *C. elegans*, fruit flies, and zebrafish have an identified wild type in addition to the different genetic breeds with different characteristics that have been developed for research purposes. In stark contrast, the vastly different chicken breeds have existed for so long (at least in terms of biomedical developmental research) that it is very difficult to trace their origins. This lack of a clearly identified wild type, paired with the aberrant biology mentioned above and the always present possibility of unknown pleiotropic genetic effects, suggest that biomedical researchers are using a “humanly fabricated” animal.

### Comparing Chickens to Other Birds: Verifying the Model

Animal models, as indicated above, have contributed enormously to almost all aspects of biomedical research, and certainly avian models—especially the chicken—have played an important role in this advancement. However, as any animal model becomes more popular, the tendency to question its ongoing validity naturally wanes. Yet, questioning animal model validity should be an on-going process. Consequently, here we ask the questions: “*Should we periodically be validating (re-validating) the chicken model as being sufficiently representative of other avian and non-avian species”?* and, if so “*What would such validation look like?*”

**Figure 3:**
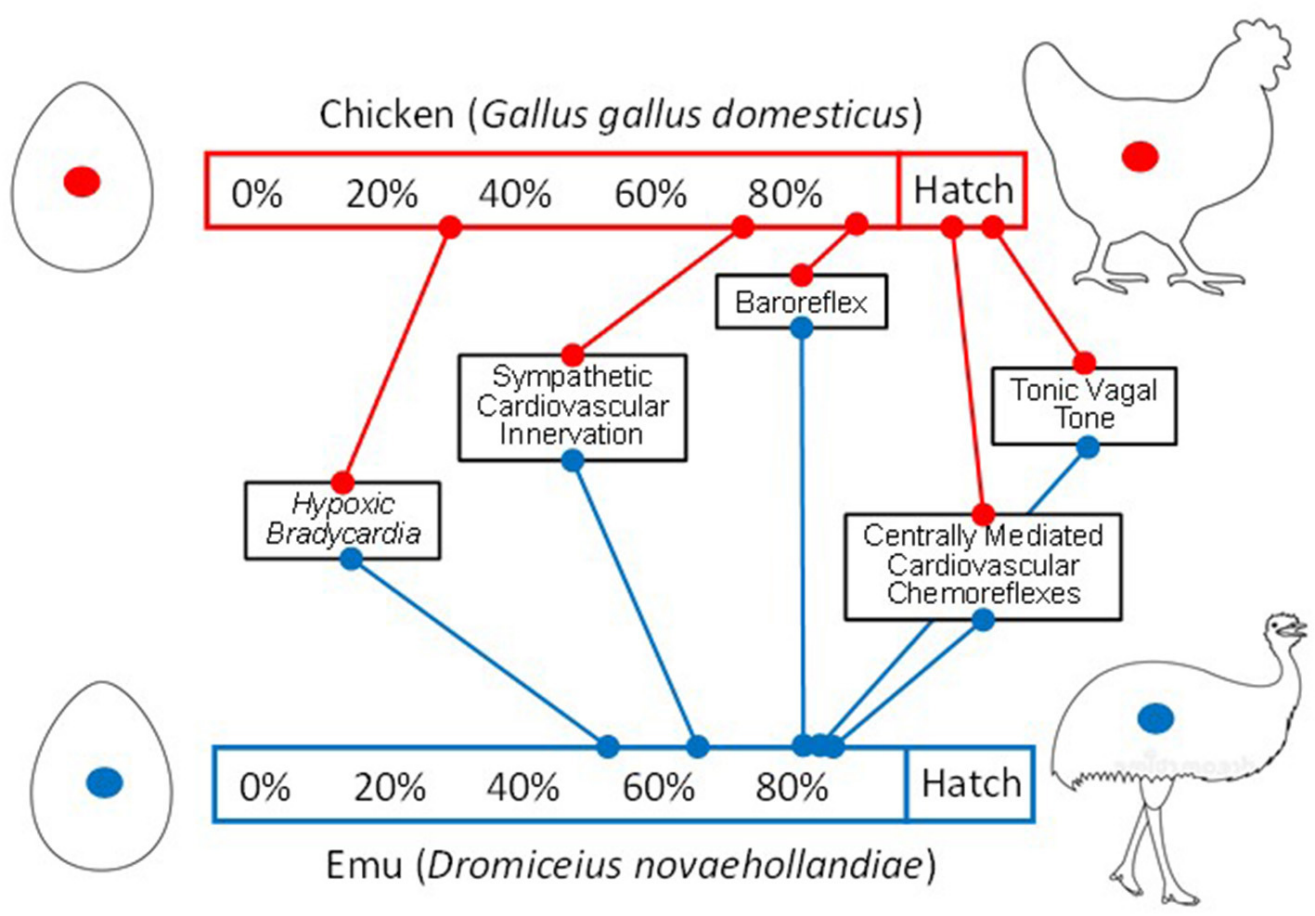
A comparison of the onset of elements of cardiovascular control in the developing embryo of the domestic chicken and the emu. Development has been normalized to 100% of development, followed by a hatching period. Note that major developmental landmarks in the emu occur later (e.g., hypoxic bradycardia) or earlier (e.g., onset of tonic vagal tone) than in the domestic chicken. This raises the question “Which species is ‘representative'?" (modified from Crossley et al., 2003; Dzialowski and Greyner, 2008).

Much has been written on the rationale and process of animal model validation (e.g., Krebs, 1975; Strange, 2007; Heston and White, 2017; Robinson et al., 2019) and it is not our intention to revisit this topic in any detail. However, with the heavy reliance on the chicken in developmental physiological research, we do suggest that parallel experiments in other avian species be performed from time to time to verify the translational value of the acquired data. As an example of the relevance of multiple model comparisons, consider the extensive use of the chicken embryo as a model for the ontogeny of vertebrate cardiovascular control (Vleck et al., 1979; Ricklefs, 1985; Gillespie and Schupp, 1998; McNabb, 2006; Andrewartha et al., 2011; Dzialowski et al., 2011; Shell et al., 2016; Scheiber et al., 2017; Tobalske et al., 2017; Price and Dzialowski, 2018; Ruaux et al., 2020). Basic aspects of the development of neural and endocrine regulatory elements of birds are presumed to map onto the mammalian and even the human condition, at least in general terms. However, this unquestioning use of the chicken embryo, juvenile and adults begs the question “*Do findings from chicken ontogeny even map onto the overall avian condition, let alone that of other vertebrate classes*”? To emphasize this point, Figure 3 compares the development of cardiovascular control in the chicken and emu embryos, normalized to the length of the incubation period. In this case of evolutionary heterochrony, several key developmental landmarks in the ontogeny of their cardiovascular regulation differ substantially between the chicken and the emu. This leads us to ask “*Is it the chicken or the emu that is the ‘representative' avian species when it comes to cardiovascular development*?” or even “*Is there a representative avian* species?”

In another sphere of cardiovascular-based biomedical research, the vessels of the chicken embryo's choriallanotic membrane (CAM) have been used as a model vascular bed for angiogenesis (Ricklefs, 1985; Schew et al., 1996; McNabb, 2007; Wada, 2008; Bateson and Feenders, 2010; Margoliash, 2010; Ottinger et al., 2013; Nowak-Sliwinska et al., 2014; Olson et al., 2014; Bolhuis and Moorman, 2015; Aldhafiri et al., 2019; Bertram et al., 2020). The CAM is a highly specialized vascular bed for gas exchange generally viewed as homologous to the fetoplacental vessels of mammals. Yet, a strict assessment of the evolution of the placenta suggests that these vessels are analogous rather than homologous to those of the placenta (Lovell et al., 2011; Clayton and Emery, 2015; Fishbein et al., 2020), and perhaps not even equivalent to any “regular” pulmonary or systemic vascular bed of the chicken embryo or mammalian fetus. Moreover, we know little about CAM differences between birds with different taxonomies, habitats, size, and other traits. Validation of the CAM of the chicken embryo by comparison with other avian species is warranted, including a characterization of this vascular bed's pharmacological, physiological, and morphological traits by comparing them to chicken systemic vessels and to those vessels of mammals for which the CAM vessels are being used as a model.

We propose that, ultimately, experiments specifically designed to validate the chicken as a model will either strengthen its role (likely) or perhaps less likely but equally importantly, lead to additional, more appropriate models.

## Alternative Avian Models for Research in Development

Before suggesting non-chicken avian models potentially useful in biomedical research, it is important to first consider what characteristics embody a useful animal model candidate. Bolker (Koslová et al., 2020) has distilled this down to two factors: “convenience” and “convention.” However, from these two broad categories can be extracted several specific attributes, each of which can be important in weighing the merits of an alternative animal model, as we now consider.

### What Makes an Effective Bird Model for Developmental Research?

#### Accessibility

An animal model is typically readily available rather than difficult to acquire. This allows widely distributed research communities to participate in experimental verification and data replication. For example, the fruit fly *D. melanogaster* and the nematode worm *C. elegans* are maintained in biological laboratories throughout the world, which has continued to add to their popularity as an animal model. Notwithstanding the value of more exotic animal models as prescribed by the August Krogh principle, this characteristic of accessibility is a primary factor that leads to the building of research communities around single species. So, exotic birds have led to important findings, such as showing that birds can sleep while flying by studying the brain wave patterns during flying of the large, long-term flier the great frigate bird (*Fregata minor*) in the Galapagos Islands. Yet, the greater frigate bird is hardly likely to become a true animal model due to the difficulty of access to these birds (Rattenborg, 2017).

#### Rearing

A key property of an animal model is its ease of rearing. An animal may have useful characteristics for animal experimentation but is unlikely to be exploited as a model in developmental research unless it can be easily reared at minimal cost and effort. Thus, small birds like zebra finches (*Taeniopygia guttata*) are increasingly being used as models for understanding the inheritance and development of communication (Mello and Clayton, 2015; London, 2020), mainly because they are relatively easy to maintain in captivity, are relatively fecund, and the eggs can be hatched and reared.

#### Size

Just as small size was evoked above as an advantage for rearing, large size can be an important attribute for some experiments, especially in developmental studies where large eggs and their large embryos can be a tremendous advantage. Thus, the ratites (ostrich, rhea, emu, cassowary, and kiwi) lay relatively large eggs that have been used widely in developmental studies specifically because of their large embryos (Lewis et al., 2013; Caudill et al., 2015; Kelly et al., 2018; Whitney and Cristol, 2018) [see section Ratites (Emu, Ostrich, Rhea) below].

#### Fecundity

Related to the characteristics of accessibility and rearing is fecundity—the reproductive output of the species. A bird species can have numerous advantageous characteristics, but if it is a seasonal layer with small clutches, then the availability to carry out replications, etc., could be minimal. Doves and hummingbirds, for example, only produce a clutch of one or two eggs a few times in their breeding season, a characteristic that may overshadow any interesting properties as a model that they might have. It is this feature of high and steady fecundity that has driven avian-based developmental research so strongly to domesticated birds like the chicken.

#### Development and Growth Rates

**Figure 4:**
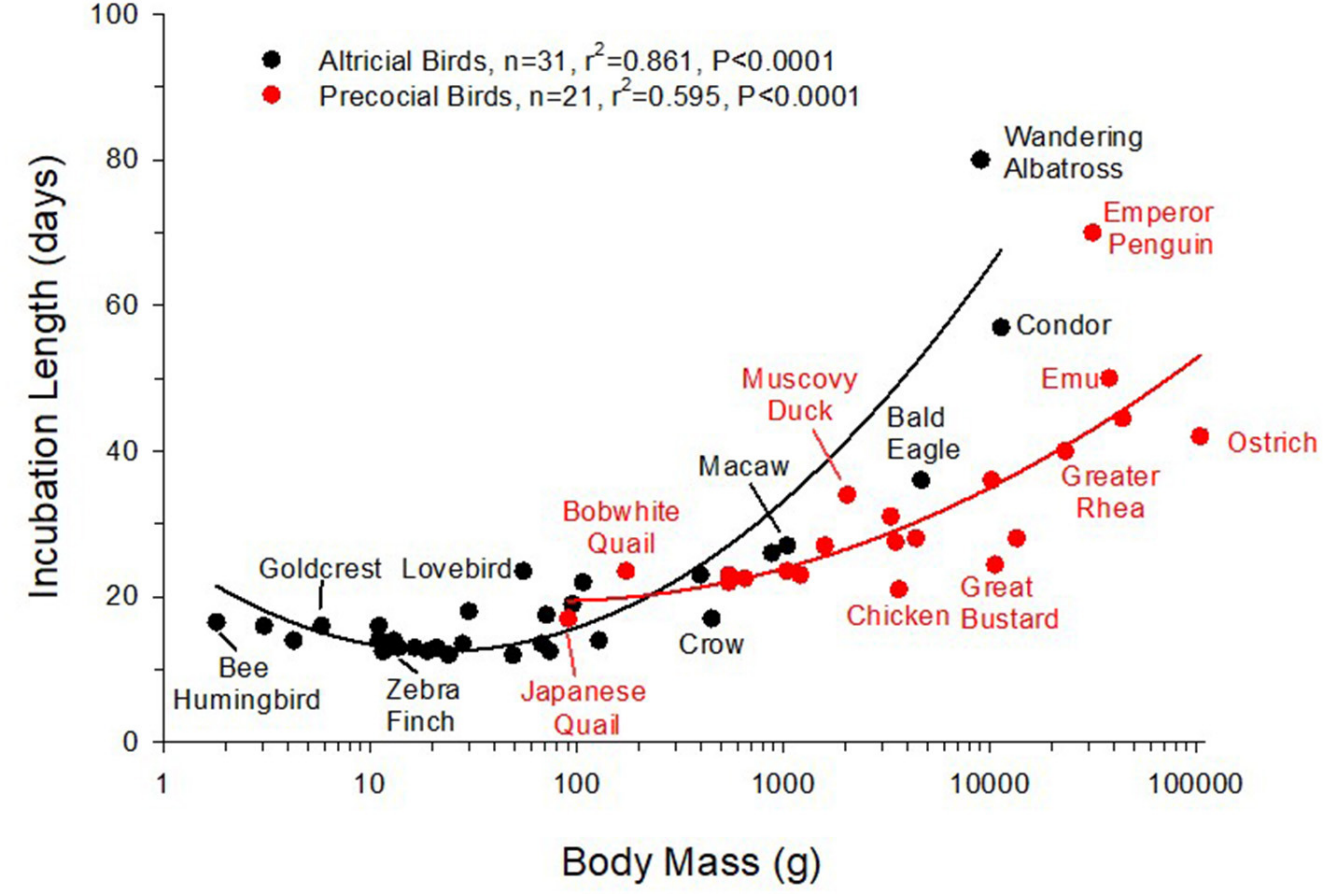
Relationship between body mass and egg incubation length in altricial and precocial birds. Body masses are the average for the species. Second order linear regressions are shown separately for precocial birds (black lines) and altricial birds (red lines).

Incubation lengths vary widely in bird species, ranging from under 2 weeks in small songbirds up to nearly 11 weeks in birds like the emperor penguin (*Aptenodytes forsteri*) (Figure 4). Relatively short incubation times and rapid growth rates to sexual maturity can be advantageous in avian models. Generally, shorter incubation times coupled with rapid maturation are favored because of the reduction in animal husbandry and especially the saving of time in performing experimental protocols. Species that rapidly reach sexual maturity, such as the king quail (*Coturnix chinensis*) which matures in as little as 4 weeks after hatching, are favored for transgenerational studies (e.g., epigenetic inheritance). On the other hand, large birds lay large eggs, but they tend to take longer to hatch (Figure 4). The appropriate avian model for developmental work thus emerges from the balance between incubation length and embryo size.

#### Genetic Manipulation

Increasingly, on metric of the value of an animal model will be the extent to which specific tools are available for affecting specific manipulations of its genome. Particularly exciting is the actual editing of the avian genome using lentiviral, adenoviral, electroporation, and CRISPR/Cas9 gene editing, which will further enhance the growing list of genetically modified birds (McGrew et al., 2004; Kobayashi et al., 2015; Véron et al., 2015; Sid and Schusser, 2018; Chojnacka-Puchta and Sawicka, 2020; Lee et al., 2020; Park J. S. et al., 2020; Park J. W. et al., 2020; Khwatenge and Nahashon, 2021). These technologies are allowing phenotype modification meeting both enhanced production goals in domesticated birds and creating modified birds for basic exploration of biological systems during development and in adulthood (Chojnacka-Puchta and Sawicka, 2020). Doubtlessly, the chicken genome has to date been the major focus of these technologies, but primordial germ cell cultures are available for the duck, goose, turkey quail, and pheasant, and experimentally altered phenotypes in these species are emerging from the use of gene editing (Bo et al., 2016; Chen et al., 2019; Lee et al., 2019; Park J. W. et al., 2020).

The great increasing availability of numerous molecular tools that can be exploited in avian research (Burgess, 2004; Burt, 2004a,b; Lawal and Hanotte, 2021).

### Alternative Species

Having indicated some useful general attributes of ideal non-chicken models, we now suggest some species that practically could be used in developmental research alongside of, or even in lieu of, the chicken animal model.

#### Quail

The term “quail” groups birds from two families: Phasianidea (old world quail) and Odontophoridae (new world quail). Both families present several characteristics listed in section What Makes an Effective Bird Model for Developmental Research? that makes them suitable for biomedical research. The rapid generational time is a major factor when considering quail for laboratory use, and accounts in part for the increase in the use of quail in biomedical research. The advantage of a small birds with precocial chicks that easily adapt to artificial environments should encourage researchers to consider this species. The widespread availability and constant egg production place the quail in a very close competition to the chicken. Another advantage is that under anesthesia and during surgery, the cardiovascular system of the quail is less fragile than the chicken's, and can be stressed in ways that can reveal new insights beyond what chicken could allow (Flores Santin, 2016). The bobwhite quail (*Colinus virginianus*) has been used to describe hematological differences throughout development and between sexes, and show an almost negligible hematological response to hypoxic incubation (Flores-Santin et al., 2018). The bobwhite quail has also been used to evaluate cardiovascular changes associated with fetal programming through hypoxic incubation. The evaluation through histology and pressure volume loops indicates impaired arterial relaxation in arterial ring preparations (femoral and carotid) in response to sodium nitroprusside (SNP) and acetylcholine(Ach) (Flores Santin, 2016). The bobwhite quail has also featured prominently in toxicological studies—e.g., effects of endocrine-disrupting compounds and popular medications (Touart, 2004; Bussière-Côté et al., 2016).

Rapid development to sexual maturity combined with high egg production has facilitated transgenerational studies examining the persistence of epigenetic changes in the Japanese quail (*Coturnix japonica*). Exposure of the parental generation *in ovo* to genistein (a naturally occurring isoflavin) resulted in reproductive changes that were still observable in the third generation (Leroux et al., 2017). The quail has also been successfully used to apply CRISPR-CAS9 to modify feather coloration without assimilation of adenovirus vector or mutations (Lee et al., 2019). In considering the quail for biomedical developmental research, it is important to also point out important disadvantages that could be found. For instance, the quail egg is considerably smaller than that of the chicken and therefore harder to apply instrumentation to (e.g., leads for electrocardiogram) and to experimentally manipulate. Also, some eggs like Japanese quail are mottled and brown, making it nearly impossible to candle the eggs to determine embryo viability or the location of chorioallantoic blood vessels. Some species of quail (e.g., king quail) are prone to high levels of stress from manipulation and instrumentation, potentially altering hormonal or hematological measurements influencing both adults and their offspring.

#### Ratites (Emu, Ostrich, Rhea)

The extant ratites (ostrich, emu, cassowary, rhea, kiwi) have proven to be useful animal models for developmental research primarily because of the large size of their embryos, thus embodying the concept of “gigantism” that enables experimental procedures not possible with smaller animals (Burggren, 1999, 2020). For example, a newly hatched emu (*Dromaius novaehollandiae*) or ostrich (*Struthio camelus*) is nearly the size of a juvenile or adult chicken, respectively. This single property—extraordinary size—has allowed “keyhole surgery” through an opening in the egg for investigating embryonic physiology and the transitions associated with the onset of pulmonary respiration (Steyaert et al., 2016). Thus, experiments have been conducted on late incubation emu eggs, whereby major embryonic vessels are chronically cannulated for blood pressure measurement and microsphere injection to determine intra- and extracardiac shunts (Steyaert et al., 2016). The size of the emu egg overall also allows the air cell to be cannulated and through-flow ventilated with experimental gases, thus controlling the internal egg environment when the embryo internally pips and takes its first breath. The large size of ratite embryos also makes for more tractable *in vitro* perfusion of the central vasculature in, for example, studies of their physiological and pharmacological properties as embryos. This especially the case for relatively small structures that nonetheless are of great physiological significance, such as the ductus arteriosus (Lewis et al., 2013; Steyaert et al., 2016; Jimeno et al., 2019).

To balance enthusiasm for ratites, on several fronts they are not necessarily “convenient,” to echo Bolker's words (Bolker, 2012). For example, obtaining adults and their eggs is currently not particularly easy (at least in North America), compared to the 1990s when emu production was emerging as a potentially profitable agricultural enterprise (Jacobs et al., 2014) (turning out to be erroneous given poor marketplace acceptance). Even if ratites can be acquired, their care and maintenance is not trivial. Moreover, the developmental researcher has to judge whether the potential advantages of working with large ratite embryos outweigh the risk that even the most robustly conducted experiments will be marginalized by other researchers unfamiliar with ratites as animal models.

#### Birds on the Precocial-Altricial Gradient

**Figure 5:**
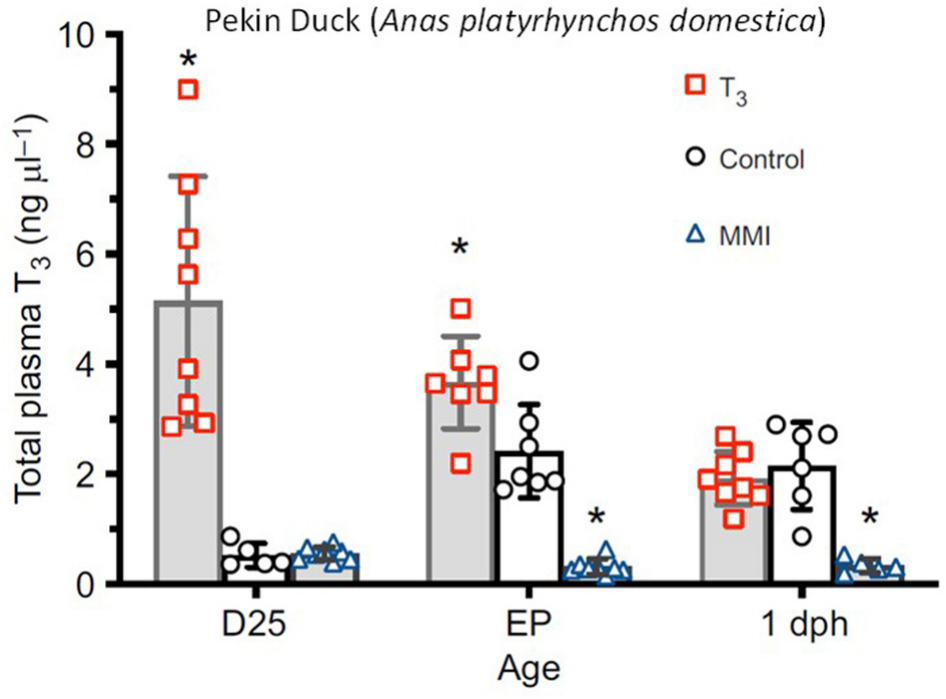
Manipulation of plasma thyroid hormone (T_3_) levels in the Pekin duck (*Anas platyrhynchos domestica*). T_3_ levels were changed by either injection of T3 or by suppression of its synthesis by injection of the thyroid-peroxidase inhibitor MMI. Total plasma [T_3_] rises from almost undetectable at embryonic day 25 (d25) to ~2 ng^.^μl^−1^ at external pipping (EP) and 1 day posthatch (1 dph). Injection of T_3_, however, elevates total plasma [T_3_] at D25 and EP. MMI strongly suppresses [T_3_] at all examined stages (from Holmes and Ottinger, 2003). *Statistically significant difference.

All birds fall at some point on a gradient comprising the characteristics Precocial, Semi-precocial, Semi-altricial and Altricial (Rutz et al., 2018). Precocial birds, which include chickens, are those birds that upon hatching are immediately mobile and have their eyes open, capable of foraging for food, and can thermoregulate within a few days of hatching. In contrast, altricial birds are born immobile with eyes closed, and require extensive parental care for days or weeks until sufficiently mature to survive on their own. Birds showing various degrees of precocial biology have been used in many studies including the ontogeny and evolution of endothermy (van der Vaart et al., 2012; Rutz et al., 2018; Cabrera-Álvarez and Clayton, 2020), adaptive plasticity (Grodzinski and Clayton, 2010), flight (Clayton et al., 2007; Clayton, 2015), and muscle growth (Salomons et al., 2009). Perhaps the precocial-altricial gradient is most relevant to biomedical research with respect to endocrine regulation (Kelleher et al., 1962; Dinsmoor, 1985; Grodzinski and Clayton, 2010; Grasman et al., 2011; Boonekamp et al., 2014). Figure 5 shows assessment and manipulation of the hormone triiodothyronine (T3) in the Pekin duck (*Anas platyrhynchos domestica*). Experiments that manipulate hormone concentrations (especially T3) in various species along the precocial-altricial gradient allow greater understanding of the role of the endocrine system in the development of endothermy, oxidative stress, reproductive organization, etc. (Skinner, 1948; Holmes and Ottinger, 2003; Grasman et al., 2011; Boonekamp et al., 2014; Burwitz et al., 2020).

Certainly, continued research with the highly precocial chicken will contribute to endocrinology and other studies but is only put in context when additional studies are carried out in other species at different points along the precocial-altricial spectrum.

#### Songbirds

**Figure 6:**
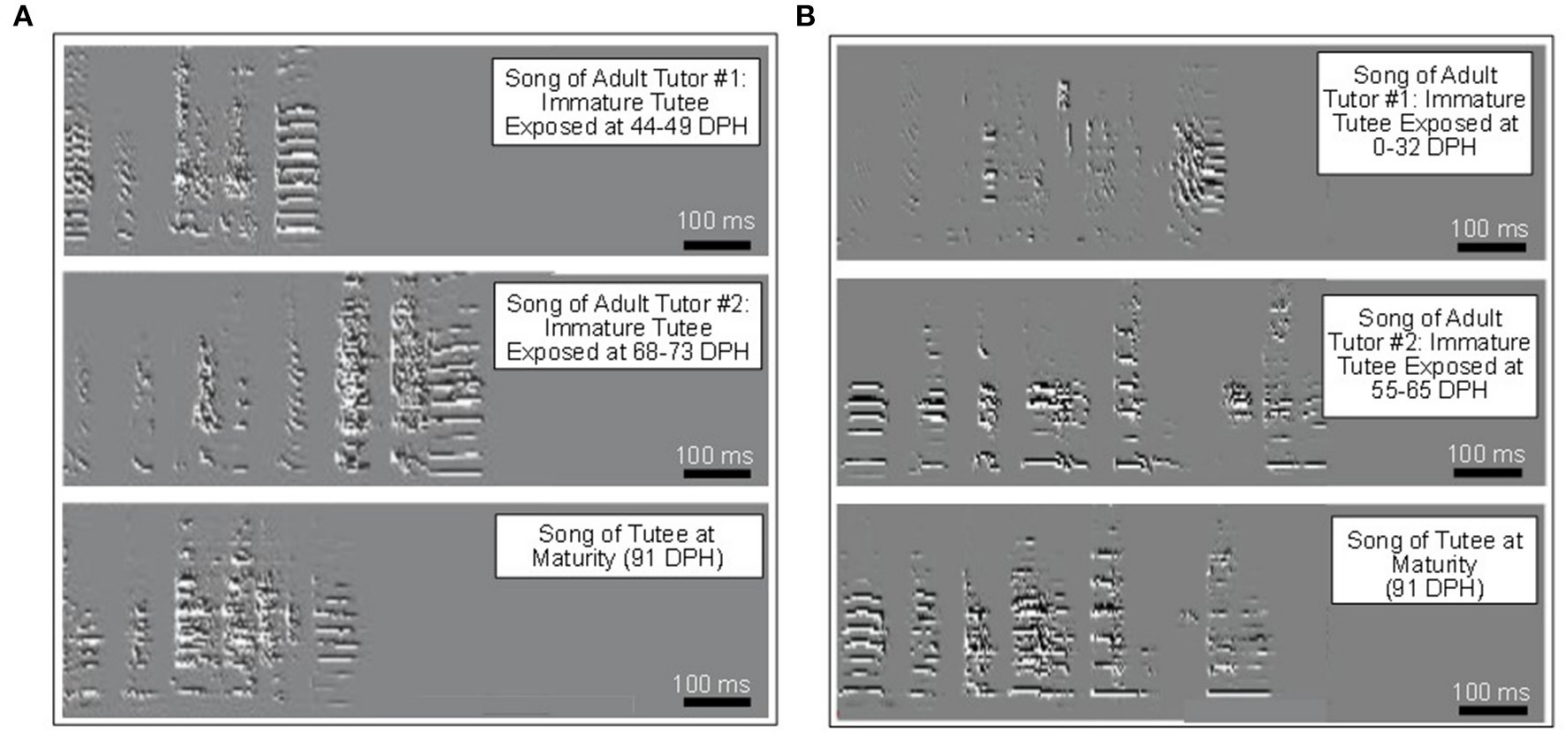
Sonograms reflecting song learning and the influence of critical windows (sensitive periods) in the zebra finch, *Taeniopygia guttata*. By exposing developing birds (the tutee, or “student”) to adult song tutors during different periods of the tutee development, the critical window for song acquisition can be identified. In **(A)**, there was an 86% resemblance to Tutor #1, but only a 57% resemblance to Tutor #2. In **(B)**, using a different pair of tutors and different tutee, there was a 64% resemblance to Tutor #1, but an 84% resemblance to Tutor #2. Collectively these and other data from such studies suggest that auditory memory forms primarily from 25 to 35 days, but that the critical window duration varies upon the interplay of songs from different tutors, particularly in the latter part of the critical window. For most immature zebrafish, the critical window “closes” after 65 days post-hatch (DPH) (modified from Gobes et al., 2019).

Passerine birds (songbirds) comprise ~4,000 species, which is nearly half of all bird species. Estimates are that up to 300,000 such individual birds are used annually in biomedical and other forms of research (Bateson and Feenders, 2010). A key focus of research using songbirds involves various aspects of learning during development that capitalizes on the development of their complex and readily quantified vocalizations. A key model in this regard in the zebra finch, which has been used to further understand the neurobiology of learning (Figure 6) (Heston and White, 2017; Gobes et al., 2019; London, 2020). The zebra finch has also been used to determine how substances commonly abused by humans alter learned song (Lovell et al., 2011; Olson et al., 2014; Aldhafiri et al., 2019), and cognition (Clayton and Emery, 2015; Fishbein et al., 2020). Such experiments have not only focused on their whole animal behavior, but also moved into assessment of the genomics, transcriptomics, and proteomics of learning (Lovell et al., 2011; Clayton, 2013; Mello and Clayton, 2015; London, 2020). Additionally, zebra finches and other passerines have been featured in ecotoxicological studies (Lewis et al., 2013; Caudill et al., 2015; Whitney and Cristol, 2018). One of the newer fronts that has opened up in songbird animal models is that of the transgenerational epigenetic inheritance of learned behaviors, especially vocalizations, and the underlying molecular mechanisms (Steyaert et al., 2016; Kelly et al., 2018; Jimeno et al., 2019). The advantage of zebra finches and other similar passerines used as animal models in such epigenetic studies is not so much that they reach sexually maturity rapidly (not compared to the king quail, for example—see above), but rather because the animal husbandry of small passerines across multiple generations is less complex, costly, and space-consuming than for other avian models.

#### Birds of Prey

**Figure 7:**
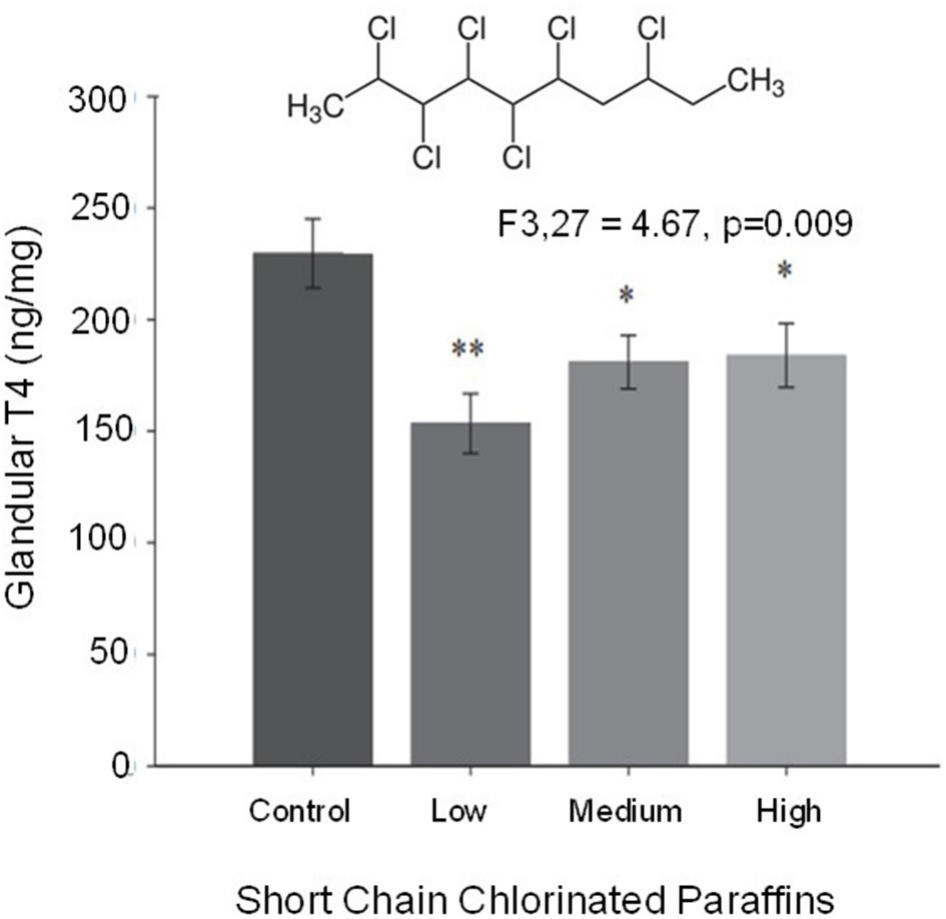
Thyroid gland concentrations of T4 from hatchling American kestrels exposed to a range of concentrations of short chain chlorinated paraffins. These compounds, an example of which is shown at the top of the graph, act as endocrine disruptors affecting thyroid function. The widely used short chain chlorinated paraffins and their high environmental prevalence constitute a threat for fauna and humans alike that could be monitored through raptors (modified from Fernie et al., 2020). *Statistically significant different group 1. **Statistically significant different group 2.

The birds of prey comprise three orders: Accipitriformes, Falconiformes, and Strigiformes. Although all are predatory birds, the presumably differ markedly in their evolution and likely in aspects of their physiology, including developmental physiology. Yet, because of their position in common at the top of many food chains, birds of prey have been studied in many contexts, perhaps most employed as models for processes of bioaccumulation of toxicants. As an example, short chain chlorinated paraffins (SCCP's), chemicals used in the metal and plastic industry, have made their way into raptors by means of ingestion. The effects of these chemicals has been evaluated in American kestrel hatchlings, where they result in impaired thyroid function and endocrine disruption (Figure 7) (Fernie et al., 2020). As another example, the tolerance to cryopreservation of sperm from golden eagle and peregrine falcon reveals different tolerances for each species providing an insight on sperm biology and how its physiology is not general even among the same group (Aves) (Blanco et al., 2000). The eyes of raptors have been analyzed, revealing visual mechanisms that have been applied to the field of optics (Snyder and Miller, 1978). As for other non-chicken bird models, research in birds of prey is being facilitated by advances in understanding of their genomics and transcriptomics (Bartholomew and Tucker, 1963; Zhan et al., 2013; Pan et al., 2017; Kang et al., 2018; Cho et al., 2019; Doyle et al., 2019; Adawaren et al., 2020). Interestingly, modern day falconry (training and hunting with raptors) offers an excellent opportunity for research on topics that require conditioning or problem-solving skills in non-mammalians, including the development of such skills. On the other hand, birds of prey are always non-domesticated birds that might not respond well to experimental handling and manipulation. Additionally, many raptor species are threatened or endangered, making their use in physiology somewhat problematic.

#### Corvids

The covidae—crows, ravens, jays, and magpies—comprise a family of birds that have been studied for their intelligence, memory, and problem solving. While not particularly convenient to house and maintain, their behavior has nonetheless been studied not only because they are an inherently interesting avian family, but also because they can serve as animal models for understanding human cognition and its evolution (Jacobs et al., 2014; Mello and Clayton, 2015). One of the key behavioral aspects upon which research has focused has been the use of tools by corvids (Rutz et al., 2016, 2018). Indeed, the convergent evolution of tool use in birds and mammals has led investigators to explore neural networks in common with both groups of animals (Cabrera-Álvarez and Clayton, 2020). Another unusual behavior of corvids is their use of object caching (Grodzinski and Clayton, 2010; van der Vaart et al., 2012; Jacobs et al., 2014). Study of the developmental and evolutionary aspects of this behavior have contributed to our understanding of human social cognition (Clayton et al., 2007) and cognitive development in children (Clayton, 2015). As a final example of the use of corvids as animal models, behavioral and cellular correlates of aging have been examined in the context of telomere length (Salomons et al., 2009; Grasman et al., 2011; Boonekamp et al., 2014). All of these categories of study are further enabled by the advances in the genomics and transcriptomics of corvids (Poelstra et al., 2015; Morinha et al., 2017; Dussex et al., 2021).

#### Other Avian Models

It's beyond the scope of this perspective (or perhaps any article) to review all bird species used in biomedical research, but there are some “honorable mentions.” Pigeons (*Columba livia*), of course, have a venerable place in behavioral research, dating back to the mid-twentieth century (Skinner, 1948; Kelleher et al., 1962; Dinsmoor, 1985). Budgerigars (*Melopsittacus undulatus*), canaries (*Serinus canaria domestica*), European starlings and house sparrows have shown promise in studies of development, aging, and energetics (Holmes and Ottinger, 2003; Austad, 2011). The duck (*Anas platyrhynchos*) has frequently been used in been featured in biomedical research, especially in studies of infectious agents including influenza (Meade et al., 2017; Burwitz et al., 2020) and in toxicology. Indeed, numerous waterfowl species beyond the duck have been investigated in the context of their toxicological responses (Mateo et al., 2003; Finch et al., 2012; Valverde-Garcia et al., 2018). Penguins (Family: Spheniscidae) have shown a number of different physiological mechanisms during diving when compared to mammals (Degernes, 2008; Mattern et al., 2018).

#### Choosing Bird Models According to Their Environment

An alternative, useful approach to choosing an appropriate avian model for investigating a particular research question by its species involves selections based on a particular environment. Thus, investigations of thermoregulation, either could employ desert birds or those inhabiting polar regions (Blix, 2016; McKechnie et al., 2021). These avian models could allow a better understanding of both temperature stress in domestic species, as well as basic questions like the development and evolution of endothermy (Dzialowski et al., 2007; Price and Dzialowski, 2018; Perini et al., 2020; Goel, 2021; Kpomasse et al., 2021). Similarly, understanding of chronic oxygen deprivation associated with high altitude and its effect on development and other processes can be facilitated by physiological and genetic investigations either species or breeds that normally inhabit high altitude, or by laboratory exposures to hypoxia (Dzialowski et al., 2002; Chan and Burggren, 2005; Zhang and Burggren, 2012; Burggren and Elmonoufy, 2017; Zhang et al., 2019, 2020; Tang et al., 2021). Water relations during development can also be exposed by comparing desert birds with congeners inhabiting less extreme environments. Thus, the Gray gull (*Larus modestus*) inhabiting the Atacama Desert, one of the driest places in the world, has an eggshell gas permeability only about 1/3 that of other species of *Larus*. This lower permeability reduces water vapor loss, but also inhibits inward oxygen diffusion, resulting in lower oxygen consumption and much longer incubation time in the Gray gull compared to other gulls. These experiments reveal the relationship between gas diffusion, egg shell conductance, and development in a model chosen for its location, rather than its taxonomy (Monge et al., 2000).

## Discussion: Conclusions and Future Directions

As indicated at the outset of this article, our intent has not been to discredit the chicken as an animal model, but rather to urge exploration of additional models that can enhance our understanding of developmental physiology and other disciplines. To this end, we offer several suggestions for future research.

### Verifying the Chicken Model

**Figure 8:**
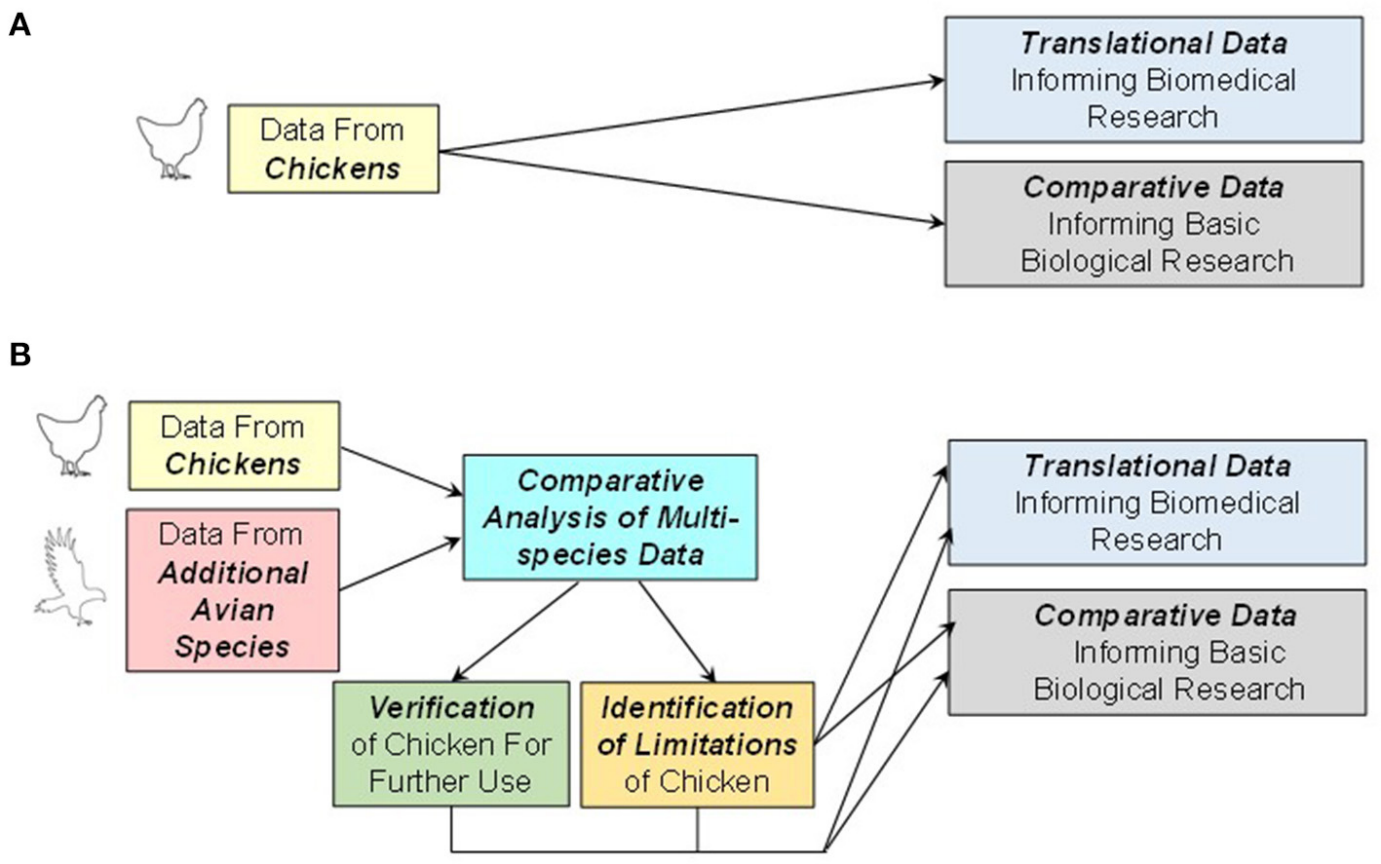
Traditional and proposed use of developmental data acquired using the chicken model. **(A)** In many studies, data acquired from chickens are used to inform both basic biological research as well as biomedical research. These data are assumed to be representative of all birds and are often accepted without question. **(B)** An alternative approach that incorporates data from both chickens and additional avian species will enable comparative analyses to determine the appropriateness of the animal model employed. Such analyses can either verify the chicken as an animal model or identify limitations of data derived from the chicken that could be overcome by expanding research to include alternative avian models.

We advise that, when carrying out developmental research using the chicken as an animal model, data be collected from additional avian species where practical to validate the core translational value of the chicken data (Figure 8). In situations where specific protocols and techniques have been developed around *G. gallus domesticus*, it may be possible to turn into an asset the complex genetics of the chicken that has resulted in several highly selected breeds. Thus, identifying the genetic underpinnings of some of the divergent (and in some cases aberrant) biological characteristics of various breeds may yield greater overall insight than studying one chicken breed, alone.

### Expanding Beyond the Chicken as a Biomedical Models

While the chicken remains an undisputed powerful, useful, and practical model in developmental biomedical research, there are many additional, and possibly alternative, bird models that could further elucidate key biological mechanisms and responses. When exploring alternative avian models, proposed studies should of course carefully evaluate the suitability of the proposed model. An excellent guideline to this process, derived from the field of toxicology, has been offered by Jaspers (2015). Important is to recognize the possible shortcomings of the chicken, even as being aware of its advantages. Once the strengths and weaknesses of the chicken as a model are appreciated, then research in developmental physiology and other areas will be strengthened through a creation of a “search image” for additional avian models.

### Identifying Novel Avian Models

Doubtlessly, there are additional bird species with novel characteristics that will help advance not just avian research, but in vertebrates including man, generally. It is for those of us in developmental and forms of research to identify them—the rewards are potentially great. After all, who had heard of the zebrafish before the work of George Streisinger or the Tubingen/Boston mutant screens, or of *C. elegans* before Sidney Brenner's seminal 1974 paper (Brenner, 1974; Hörstgen-Schwark, 1993). The opportunity to expand the base of avian biomedical research has never been greater.

## Data Availability Statement

The raw data supporting the conclusions of this article will be made available by the authors, without undue reservation.

## Author Contributions

All authors listed have made a substantial, direct and intellectual contribution to the work, and approved it for publication.

## Funding

We acknowledge financial support from the US National Science Foundation (#IOS-1025823) and CONACYT for the SNI professor stipend.

## Conflict of Interest

The authors declare that the research was conducted in the absence of any commercial or financial relationships that could be construed as a potential conflict of interest.

## Publisher's Note

All claims expressed in this article are solely those of the authors and do not necessarily represent those of their affiliated organizations, or those of the publisher, the editors and the reviewers. Any product that may be evaluated in this article, or claim that may be made by its manufacturer, is not guaranteed or endorsed by the publisher.
